# F10 Gene Expression and Ethnic Disparities Present in Papillary Thyroid Carcinoma

**DOI:** 10.3390/jpm14050524

**Published:** 2024-05-15

**Authors:** Tyrel Porter, Lilia Kucheryavykh

**Affiliations:** Department of Biochemistry, Universidad Central del Caribe, Bayamón, PR 00956, USA; lilia.kucheryavykh@uccaribe.edu

**Keywords:** papillary thyroid carcinoma, ethnic disparities, factor X, F10

## Abstract

Papillary thyroid carcinoma (PTC) presents a significant health concern, particularly among Hispanic women in the United States, who exhibit a disproportionately higher chance of developing an advanced disease when compared to the non-Hispanic population. Emerging evidence suggests coagulation factor X, encoded by the F10 gene, has a potential role in inhibiting cancer cell migration. However, comprehensive investigations into the differential expression patterns of F10 in Hispanic versus non-Hispanic females remain limited. RNA-sequencing data were acquired from the TCGA database for white female patients, 166 non-Hispanic and 25 Hispanic. A statistically significant (*p* < 0.05) 2.06-fold increase in F10 expression levels was detected in disease-free tumors compared to recurrent PTC tumors. Furthermore, an increase in F10 gene expression levels was also observed, corresponding to approximately a 1.74-fold increase in non-Hispanic patients compared to Hispanic patients. The probability of tumor recurrence was 1.82 times higher in the cohort with low expression of F10 compared to the high-expression cohort, correlating with the lower disease-free rates observed in the Hispanic patient cohort when compared to non-Hispanics. This finding underscores the relevance of ethnic disparities in molecular profiles for understanding cancer susceptibility. Identifying F10 as a potential prognostic biomarker highlights avenues for targeted interventions and contributes to improving diagnostic and treatment strategies for diverse patient populations.

## 1. Introduction

Papillary thyroid carcinoma (PTC) presents a significant health challenge, particularly among women, who exhibit an increased incidence rate when compared to men [1]. Although mortality rates are low relative to other types of cancer, PTC is particularly susceptible to recurrence [2,3]. Furthermore, Hispanic women in the United States have a disproportionately higher likelihood of experiencing recurrent PTC compared to their non-Hispanic counterparts [4]. However, despite this increased susceptibility to advanced disease, Hispanics paradoxically exhibit lower mortality rates [5].

Various factors have been implicated in PTC recurrence, including gender, tumor size, distant metastasis, and age [6,7,8]. Additionally, total thyroidectomy surgeries have achieved lower recurrence rates compared to partial lobectomies [9,10]. However, emerging PTC research has highlighted the prognostic potential of gene expression markers such as mRNA, microRNA, and somatic mutations [11]. Multi-omic data analyses have enabled a deeper understanding of the molecular landscape in cancer and provide valuable insights regarding risk stratification.

The coagulation cascade is closely connected to the pathogenesis of cancer and influences the components of the tumor microenvironment, exerting both direct and indirect effects [12]. Coagulation proteases, and subsequent activation of protease-activated receptors, have demonstrated mixed effects on cancer progression. However, recent studies have associated activation of these receptors with limited tumor growth as well as an important mediator in apoptosis [13,14]. Coagulation factor X (FX), a coagulation protease encoded by the gene F10, has garnered attention in recent cancer research due to its involvement in tumor progression, including PTC [15,16]. Findings from prior investigations, which demonstrate the detection of FX mRNA within cancerous tissue, suggest the potential for malignant cells to produce this coagulation factor [17]. Notably, FX has demonstrated evidence of inhibiting cancer cell migration as well, suggesting a multifaceted involvement in cancer progression [18]. 

We hypothesize that ethnic disparities in PTC incidence and prognosis may be attributed to differential expression of FX, which will be manifested in F10 gene expression. In this study we demonstrated that lower gene expression levels in F10 correlate to the increased risk of advanced disease and lower disease-free rates observed among Hispanic women with PTC compared to non-Hispanic women.

## 2. Materials and Methods

### 2.1. Gene Expression Data

Clinical data and expression values for F10 in white female patients, both Hispanic and non-Hispanic (Thyroid Carcinoma, The Cancer Genome Atlas Program (TCGA) Provisional for Cancer Genomes and mRNA expression (RNA Seq V2 RSEM)), were obtained from the cBioPortal for cancer genomics (http://www.cbioportal.org, accessed on 17 April 2024) [19,20], which contains annotated TCGA data. A papillary thyroid cancer (TCGA, Firehose Legacy) mRNA expression dataset, containing 192 samples, was directly downloaded from the cBioPortal on 29 February 2024. 

### 2.2. Statistical Analysis

RNA sequencing values were log2-transformed to model proportional changes. Unpaired *t*-tests were employed to assess the significance of differences in gene expression between groups with recurrent PTC and disease-free status. A *p*-value threshold of <0.05 was considered statistically significant. Unpaired *t*-tests were employed to evaluate significance for both gene expression and disease-free status. 

To assess overall survival, as well as the impact of gene expression levels on recurrence rates, log-rank (Mantel–Cox) tests were performed. Genes were categorized into high-expression and low-expression groups using the median and, subsequently, by using ±1.5 z-scores as a cutoff. Additionally, the Gehan–Breslow–Wilcoxon test was utilized to assess differences in survival curves between these groups. Hazard ratios (HRs) were calculated to quantify the risk of recurrence associated with high and low gene expression levels. The Mantel–Haenszel method and log-rank test were utilized to compute HRs with confidence intervals (CIs) of 95%. All statistical analyses were conducted using GraphPad Prism 9.1.0 software.

## 3. Results

### 3.1. Analysis of Gene Expression Differences in Disease-Free and Recurrent Papillary Thyroid Carcinoma

**Figure 1:**
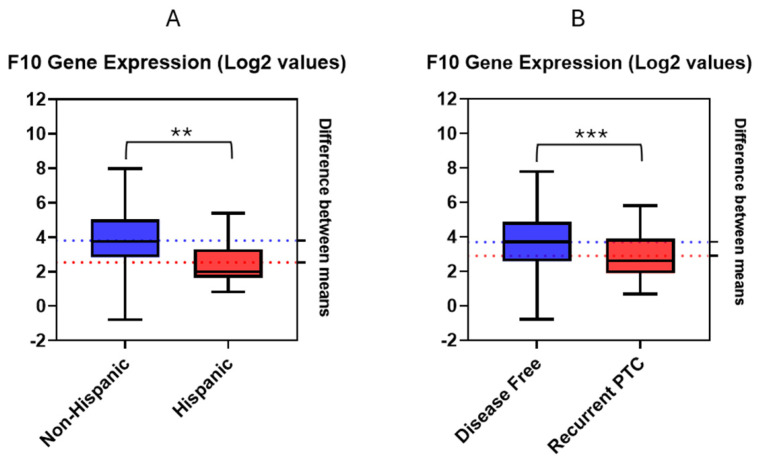
Estimation plots using log2-transformed RNA-Seq values for F10 gene expression obtained from cBioPortal. (**A**) Estimation plot for F10 gene expression values in disease-free and recurrent PTC (*p*-value = 0.001, t-value = 2.18); (**B**) Estimation plot for F10 gene expression values in Hispanic and non-Hispanic patients (*p*-value = 0.0003, t-value = 3.69). ** *p* < 0.005; *** *p* < 0.0005.

Analysis of F10 mRNA expression data without regarding any specific timeframe, by means of an unpaired *t*-test, revealed statistically significant differences in gene expression values between groups of patients remaining disease free and those experiencing recurrence (Figure 1A). Clinical data on disease status were present for 184 patients. In the disease-free group, comprising 162 patients, and the recurrent PTC group, consisting of 22 patients, differences in disease status yielded a t-value of 2.18 (*p* = 0.03). Specifically, disease-free patients showed a 2.06-fold increase in F10 mRNA expression compared to patients who experienced recurrence. 

Employing a point-biserial correlation test also yielded significant results (*p* = 0.001) between F10 expression levels and disease-free status within a 5-year period. A correlation coefficient of −0.248 was found, indicating a moderate negative correlation between F10 gene expression and the incidence of recurrence. This observation aligns with our previous findings indicating that decreased F10 expression is linked to an elevated risk of disease recurrence in PTC patients.

### 3.2. Ethnic Disparities in Gene Expression Profiles

Further analysis, by means of an unpaired t-test, was performed on the log2-transformed F10 gene expression data for the 184 living patients containing clinical information regarding disease status. These patients were subsequently divided into two ethnic groups: 25 Hispanic and 166 non-Hispanic patients (Figure 1B). A statistically significant difference in F10 expression was observed between Hispanics and non-Hispanics (*p* = 0.0003), with a t-value of 3.69. This difference corresponded to a 1.74-fold increase in F10 expression in non-Hispanic patients compared to Hispanic patients. 

A point-biserial correlation test showed a negative correlation between the F10 gene expression and being Hispanic, with a correlation coefficient of −0.169 (*p* = 0.019). This finding further supported ethnic disparities demonstrated in F10 gene expression from the unpaired *t*-test.

### 3.3. Comparison of Overall Survival Curves

Overall mortality data were limited given the positive prognosis for PTC in most cases. Of the 192 patients, only 8 were reported as deceased. However, survival analysis was performed to assess the impact of F10 expression on patient outcomes in recurrent PTC. High- and low-expression groups were defined using mRNA expression z-scores above and below the median, respectively. Both the log-rank test and Gehan–Breslow–Wilcoxon test were conducted to compare the overall survival (OS) curves between the 192 patients. Based on the analysis of overall survival, both the log-rank test and the Gehan–Breslow–Wilcoxon test yielded non-significant results. The log-rank test produced a chi-square value of 0.02086 with a *p*-value of 0.8852, while the Gehan–Breslow–Wilcoxon test resulted in a chi-square value of 0.001909 with a *p*-value of 0.9652. These values, although quite limited given the sample size, suggested no significant differences in OS curves between Hispanic and non-Hispanic patients in the accessed dataset.

### 3.4. Comparison of Recurrence-Free Survival Curves

**Figure 2:**
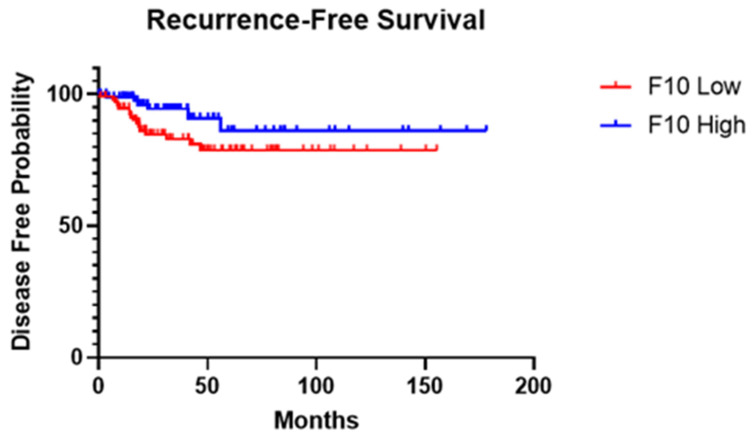
Kaplan–Meier survival analysis of PTC recurrence for high- and low-expression groups while using F10 gene expression z-score median as differentiator. RNA-Seq data analysis of 184 patients from The Cancer Genome Atlas Program (TCGA, Firehose Legacy, Papillary Thyroid Carcinoma) was used. Curve comparison was performed with use of Gehan–Breslow–Wilcoxon test: χ^2^ = 4.477, *p*-value = 0.0344.

To assess recurrence probability, patients were divided into high- and low-expression groups using mRNA expression z-scores above and below the median, respectively (Figure 2). Based on the recurrence-free survival (RFS) analysis results, there was a notable difference observed in the RFS curves between patients with low and high F10 expression levels. The log-rank test yielded a chi-square value of 3.301 with a corresponding *p*-value of 0.0692. However, the Gehan–Breslow–Wilcoxon test showed an χ^2^ value of 4.477 and a *p*-value of 0.0344, indicating a significant difference in RFS curves between the two expression groups. Furthermore, the HR analysis showed a ratio of 2.179, indicating a higher risk of recurrence in patients with low F10 expression compared to those with high expression. Conversely, the reciprocal of this ratio was 0.4590, suggesting a reduced risk of recurrence in patients with high F10 expression. Additionally, the HR calculated using the log-rank method revealed similar findings, with a ratio of 2.321 indicating a lower risk in patients with high F10 expression and a reciprocal of 0.4308 indicating a higher risk in patients with low F10 expression. 

High- and low-expression groups were further dichotomized using z-scores of ±1.5 relative to all samples. While analyzing 5-year recurrence rates, the log-rank test revealed a statistically significant difference in recurrence between patients with low and high F10 expression (χ^2^ = 5.003, *p* = 0.0253). Similarly, the Gehan–Breslow–Wilcoxon test confirmed this finding, demonstrating a significant divergence in survival curves (χ^2^ = 4.918, *p* = 0.0266). In comparison to high- and low-expression groups defined by the median, ±1.5 z-score groups yielded more significant results, further highlighting the role F10 may play in recurrent PTC. 

HRs were calculated to quantify the risk of recurrent papillary thyroid cancer associated with high F10 expression compared to low expression. The Mantel–Haenszel method yielded an HR of 14.43 (95% CI: 1.391 to 149.6), indicating a substantially elevated risk of recurrence in patients with low F10 gene expression. Conversely, the reciprocal of the hazard ratio was 0.06932 (95% CI: 0.006685 to 0.7188), signifying a markedly reduced risk of recurrence in patients with low F10 gene expression. 

## 4. Discussion

The disparities in the incidence, progression, and prognosis of cancer among different ethnic groups, particularly Hispanic and non-Hispanic populations, have long been recognized but poorly understood [21,22]. Our investigation of the distinct expression patterns suggests a decreased level of F10 expression in PTC cases increases susceptibility to recurrence. Furthermore, a similar decrease in F10 expression is evident among Hispanic patients diagnosed with PTC in comparison to their non-Hispanic counterparts. These observed ethnic disparities in F10 expression levels present a possible explanation regarding the increased rates of PTC recurrence observed in Hispanic women [4].

Our RFS results suggest the prognostic value of F10 gene expression in PTC recurrence. Utilizing the median to divide gene expression into low- and high-expression groups achieved statistical significance in the Gehan–Breslow–Wilcoxon test, with a *p*-value of 0.0344, confirming associations between low expression and recurrence. However, significance in this association was increased for both the log-rank and Gehan–Breslow–Wilcoxon tests when gene expression values between ±1.5 z-scores were excluded (*p* = 0.0253, *p* = 0.0266, respectively). The increased significance observed highlights the potential utility of F10 as a biomarker for risk stratification and treatment decision-making in PTC patients.

The associations between PTC recurrence and decreased F10 expression levels, which were also present in Hispanic patients compared to non-Hispanic counterparts, prompt critical reflections on the multifaceted roles of FX in cancer biology. Our findings align with studies performed on colon, lung, and breast cancer cells that suggest FX levels may inhibit cancer progression [18]. FX, in addition to other coagulation proteases, could plausibly contribute to reduced recurrence rates due to the association between protease-activated receptors and reduced autophagy [14,23], particularly given the association between reduced autophagy and an increased risk of recurrence [24]. Additionally, it is plausible that this link is elucidated by the role FX plays in angiogenesis. FX, as both a zymogen and its activated form, has been shown to exhibit anti-angiogenic properties [25]. Tumor angiogenesis has been identified as a risk factor for cancer recurrence and a prognostic for patient survival in multiple studies [26,27,28]. For this reason, anti-angiogenic agents have been utilized successfully for the prevention of cancer progression [29,30,31]. Speculatively, these anti-angiogenic properties demonstrated by FX may contribute to the protective role against cancer recurrence uncovered in our study.

Our study is not without limitations, however. One notable limitation is the assumption that gene expression levels directly translate into protein expression and functional outcomes. Although we observed statistically significant differences in F10 mRNA expression between patients with recurrent PTC and those remaining disease free, we did not directly measure protein expression levels or functional activity of FX in tumor progression. This discrepancy between gene expression and protein expression/function could be influenced by post-transcriptional and post-translational regulatory mechanisms, such as alternative splicing, mRNA stability, translation efficiency, and protein degradation rates. More research is needed to fully understand the mechanisms and associations, and further investigations are needed to delineate the functional significance of F10 dysregulation in PTC progression and its potential as a therapeutic target.

Furthermore, the link between reduced F10 gene expression and Hispanic patients warrants additional investigation. Racial disparities in cancer outcomes are often impacted by non-genetic factors such as access to care and sociodemographic data [32]. A deeper investigation, while accounting for these variables, may contribute to the understanding of the disparities in PTC recurrence in our study as well as confirm the prognostic value of FX gene expression.

In conclusion, our study provides compelling evidence of ethnic disparities in F10 expression patterns in PTC and highlights a potential prognostic for disease recurrence. The study revealed a relationship between low expression of F10 and the higher rates of recurrence observed in the Hispanic patient cohort compared to non-Hispanics. These findings suggest the significance of F10 as a molecular determinant contributing to ethnic variations in PTC outcomes.

## Data Availability

The data generated in this study are available within the article.
